# Maltraitance des étudiants et facteurs associés à la Faculté de Médecine de Parakou en 2018

**DOI:** 10.11604/pamj.2019.34.150.16367

**Published:** 2019-11-14

**Authors:** Djivèdé Witchékpo Maurice Mohamed Akanni, Sonia Bignon Mahoussi Gwladys Adjadohoun, Barikissou Georgia Damien, Francis Tognon-Tchegnonsi, Alexandre Allode, Alain Aubrege, Kofi-Mensa Stéphane Savi de Tove

**Affiliations:** 1 Faculté de Médecine, Université de Parakou, Parakou, Bénin; 2 Faculté des Sciences de la Santé, Université d'Abomey, Calavi, Bénin; 3 Faculté de Médecine, Université de Lorraine, Lorraine, France

**Keywords:** Comportements de maltraitance, facteurs associés, faculté de médecine de Parakou, Mistreatment, associated factors, faculty of medicine of Parakou

## Abstract

**Introduction:**

Plusieurs études à travers le monde ont montré l'importante survenue des comportements de maltraitance subis par les étudiants en médecine. Notre objectif a été d'étudier les comportements de maltraitance subis par les étudiants et les facteurs associés au sein de la Faculté de Médecine de Parakou (FM/UP) en 2018.

**Méthodes:**

Une enquête transversale descriptive et analytique a été conduite du 1^er^ au 28 février 2018 chez les étudiants de la FM/UP. Les participants étaient les étudiants en 2^ème^ année médecine (PCEM2), en 4^ème^ année de médecine (DCEM2) et en 6^ème^ année de médecine (DCEM4) inscrits au titre de l'année universitaire 2017-2018 qui ont librement donné leur consentement éclairé pour participer à l'étude.

**Résultats:**

Cent pourcent des étudiants de la FM/UP avaient subi au moins une fois un comportement de maltraitance. Les étudiants subissaient fréquemment un comportement de maltraitance dans 34,34% des cas. L'humiliation, la violence verbale et le fait d'être amené à effectuer un service personnel étaient les formes de violences les plus subies. Cependant, environ 10% des étudiants ont subi un harcèlement sexuel. Les auteurs de ces comportements de maltraitance subis par les étudiants étaient d'abord les médecins/enseignants, et les infirmiers puis les internes. Les étudiantes étaient trois fois plus harcelées sexuellement que leurs camarades du sexe opposé (p=0,0069). Plus les étudiants étaient âgés et inscrits au deuxième cycle des études médicales, plus ils subissaient l'humiliation (p=0,0001 pour l'âge et p<0,0001 pour le niveau d'étude) et la violence verbale (p=0,0007 pour l'âge et p<0,0001 pour le niveau d'étude).

**Conclusion:**

Cette étude a permis de montrer que tous les étudiants de la FM/UP ont subi au moins une fois un comportement de maltraitance depuis leur inscription dans l'université. Cette étude offre l'opportunité aux responsables de l'université de mettre en place une stratégie de communication pour un changement de comportement des enseignants et encadrants par rapports aux comportements de maltraitance. L'initiative d'un registre de plaintes serait également utile pour réduire l'ampleur du phénomène.

## Abstract (en)

**Introduction:**

Several studies around the world have shown a significant increase in student mistreatments in the Faculty of Medicine. The purpose of our study was to analyze student mistreatments and associated factors in the Faculty of Medicine of the University of Parakou (FM/UP) in 2018.

**Methods:**

We conducted a cross-sectional, descriptive and analytical survey among the students of the Faculty of Medicine of the University of Parakou from 1^st^ to 28 February 2018. Participants were second-year medical students (PCEM2), fourth-year medical students (DCEM2) and sixth-year medical students (DCEM4) attending the academic year 2017-2018 and who had freely given their informed consent to participate in the study.

**Results:**

One hundred percent of students of the FM/UP had experienced mistreatment at least once. Students had frequently experienced mistreatment in 34.34% of cases. Humiliation, verbal violence and instigation to personal services were the most common types of violence. However, approximately 10% of students had experienced sexual harassment. Perpetrators of mistreatments were doctors/teachers, nurses and interns. Female students were three times more sexually harassed than their colleagues of the opposite sex (p=0.0069). The older the students were and enrolled in the second-year of the Faculty of Medicine, the more they experienced humiliation (p=0.0001 for age and p<0.0001 for education) and verbal violence (p=0.0007 for age and p<0.0001 for education).

**Conclusion:**

This study highlights that all the students of the FM/UP have experienced mistreatment at least once after enrolment in the university. Based on this study, university officials should implement communication strategies to change the abusive behavior of teachers and supervisors. A register of complaints would also be useful in reducing this phenomenon.

## Introduction

Les facultés de médecine apparaissent comme étant des milieux très hiérarchisés avec les enseignants appelés «les maîtres» au sommet et les étudiants en bas de la pyramide. Dans ce milieu, l'usage de la violence dans les relations interpersonnelles est de plus en plus préoccupant [1,2]. En 2009, le Ministère de l'Education, du Loisir et du Sport canadien a proposé une définition de la violence afin que les milieux scolaires se donnent un langage commun. La violence est donc définie dans le milieu éducatif comme étant: «toute manifestation de force, de forme verbale, écrite, physique, psychologique ou sexuelle, exercée intentionnellement, directement ou indirectement par un individu ou un groupe, et ayant comme effet de léser, de blesser ou d'opprimer toute personne en s'attaquant à son intégrité, à son bien-être psychologique ou physique, à ses droits ou à ses biens» [3]. Selon l'Association des Collèges Américains de Médecine (AAMC), la maltraitance survient lorsque le comportement montre un manque de respect pour la dignité des autres et interfère de manière déraisonnable avec le processus d'apprentissage. Cela peut prendre la forme d'une discrimination fondée sur la race, la religion, l'appartenance ethnique, le sexe, l'âge ou l’orientation sexuelle, d'un harcèlement sexuel, d'une humiliation ou d'une violence verbale [4]. Plusieurs études à travers le monde ont montré l'importance de la prévalence des comportements de maltraitance au sein des facultés de médecine ainsi que leurs conséquences négatives sur l'épanouissement des étudiants et sur leur devenir professionnel [1,2,5-7]. Aux États Unis en 2011, et au Nigéria en 2014 respectivement 64% [5] et 98,5% [8] des étudiants en médecine avaient été l'objet d'au moins un comportement de maltraitance. Les formes de violence subies étaient surtout la violence verbale et l'humiliation [1,2,5-8] La maltraitance des étudiants en médecine est problématique à la fois dans ses effets sur l'environnement d'apprentissage et ses effets potentiellement nocifs sur le bien-être psychologique des étudiants. Les mauvais traitements sont en relation étroite avec des troubles de comportement tels que l'alcoolisme, la baisse de l'estime de soi et la dépression [7,9-12]. Au Bénin, il n'existe aucune donnée sur la maltraitance des étudiants en médecine par les encadrants et entre eux-mêmes. Compte tenu de l'impact de cette maltraitance sur l'apprentissage, l'objectif de cette étude était de déterminer la prévalence des comportements de maltraitance subis par les étudiants et les facteurs associés au sein de la Faculté de Médecine de Parakou (FM/UP) en 2018.

## Méthodes

### Cadre et méthodes d'étude

#### Cadre d'étude

Une étude multicentrique a été réalisée au Bénin, au Togo et au Burkina Faso. Elle a inclus cinq universités publiques: la Faculté des Sciences de la Santé de l'Université d'Abomey-Calavi et la FM/UP du Bénin; la Faculté des Sciences de la Santé de l'Université de Lomé au Togo; l'Unité de Formation et de Recherche en Sciences de la Santé de l'Université de Ouagadougou/Pr Joseph Ki-Zerbo et l'Institut National des Sciences et de la Santé de l'Université Nazi Boni de Bobo Dioulasso au Burkina-Faso. Il est présenté dans ce travail, l'étude réalisée à la FM/UP située dans la région septentrionale du Bénin. Elle a été créée en septembre 2001 et est la deuxième Faculté de Médecine du Bénin après celle de Cotonou. La FM/UP dispose à ce jour de 45 enseignants permanents dont 15 de rang magistral. L'effectif des étudiants inscrits pour l'année universitaire 2016-2017 était de 1136.

#### Méthodes d'étude

##### Type et période d'étude

Une enquête transversale descriptive et analytique a été conduite du 1^er^ au 28 février 2018 chez les étudiants de la FM/UP.

##### Population d'étude

Les participants étaient les étudiants en 2^ème^ année médecine (PCEM2), en 4^ème^ année de médecine (DCEM2) et en 6^ème^ année de médecine (DCEM4) inscrits au titre de l'année universitaire 2017-2018.

##### Critère d'inclusion

Le critère d'inclusion était d'appartenir à l'une de ces années d'étude; être présent dans l'amphithéâtre ou le lieu de stage au moment de l'enquête et d'accepter de remplir le questionnaire.

##### Échantillonnage

Un échantillonnage aléatoire proportionnel à l'effectif réel de chaque année d'étude a été réalisé. Les effectifs des étudiants de PCEM2, DCEM2 et DCEM4 étaient respectivement de 184, 112 et 107.

La taille de l'échantillon (n) a été calculée grâce à la formule de Schwartz.

$$\left({\text{n}=\text{pqZ}}^{2}{\text{/i}}^{2}\right)$$

Une prévalence p égale à 98,5% d'étudiants ayant été victimes d'au moins un comportement de maltraitance a été considérée. Cette prévalence est le résultat d'une étude réalisée dans les universités du sud-ouest du Nigéria [8]; q était égal à 1-p, Z était égal à 1,96 correspondant à un risque d'écart-réduit α égal à 0,05, et i représentait la précision souhaitée pour les résultats.

##### Outils et méthodes de collecte de données

Un questionnaire auto-administré a été utilisé pour collecter les données quantitatives. Ce questionnaire constitue une partie de celui utilisé par l'AAMC pour évaluer la qualité de la formation dans les écoles de médecine américaines. Ce questionnaire a été traduit en français et adapté au cadre de l'étude. L'effectif des étudiants par année d'étude a été obtenu auprès du service de scolarité de la Faculté de Médecine de Parakou et a permis d'organiser au préalable le déroulement de la collecte des données. Un pré test du questionnaire a été réalisé chez les étudiants en 5^ème^ et 7^ème^ année de médecine qui n'étaient pas concernés par l'étude. Le remplissage du questionnaire a duré en moyenne vingt minutes. Les étudiants n'ont pas été prévenus d'avance de l'enquête. Dans les amphithéâtres, les fiches ont été distribuées aux étudiants de PCEM2 et DCEM2 présents vingt minutes avant le démarrage d'un enseignement et récupérées à la fin de l'enseignement. Sur les lieux de stage, les fiches étaient distribuées dans la salle de staff aux étudiants en DCEM4 et récupérées vingt minutes plus tard. Les étudiants n'ont pas été autorisés à discuter entre eux lors du remplissage des questionnaires, mais ont reçu la consigne de donner anonymement leurs réponses personnelles et honnêtes à chaque question. Les données collectées concernent les caractéristiques socio-démographiques, les comportements de maltraitance, leur fréquence de survenue, les auteurs et le rapportage de ces comportements de maltraitance à une autorité ou un responsable étudiant.

##### Variables dépendantes

Elles ont concerné les comportements de maltraitance subis par les étudiants en médecine et/ou dont ils ont été témoins au cours de leur cursus universitaire. Ces comportements de maltraitance ont été regroupés en dix catégories: l'humiliation, la violence verbale, la violence physique, la violence liée au genre, la violence liée à l'âge, la violence liée à l'ethnie/la race/la religion, le harcèlement sexuel, être amené à effectuer des services personnels, être amené à poser un acte immoral ou non conforme à l'éthique médicale.

##### Variables indépendantes

Les variables indépendantes ont concerné les étudiants et les personnes responsables des comportements de maltraitance: les données socio-démographiques des étudiants (sexe, âge, nationalité, ethnie, statut matrimonial, l'année d'étude, le financement des études, le lieu de résidence, le redoublement) et la qualification des auteurs de comportements de maltraitance (enseignant, praticien hospitalier, infirmier(ère), agents de laboratoire et paramédicaux, résidents/internes, personnels administrative de l'université, personnel administrative de l'hôpital, camarade étudiant).

##### Gestion et analyse des données

Les données ont été saisies et analysées à l'aide du logiciel EPIINFO version 7. Les variables liées aux comportements de maltraitance ont été exprimées sous forme de prévalence. La moyenne et l'écart-type ont été calculés pour l'âge. La variable «âge» a ensuite été transformée en variable catégorielle (16-20 ans, 21-24 ans et ≥25 ans). Les autres variables socio-démographiques: sexe, nationalité, ethnie, statut matrimonial, l'année d'étude, le financement des études, le lieu de résidence, le redoublement ont été exprimées sous forme de pourcentage. Les associations entre les caractéristiques socio-démographiques et les différents comportements de maltraitance (violence verbale, humiliation, violence liée au genre, violence liée à la race/religion/ethnie, violence liée à l'âge, violence physique, harcèlement sexuel, être amené à effectuer des services personnels, être amené à poser un acte immoral ou non conforme à l'éthique médicale, et les autres violences) ont été testées. Nous avons effectué une analyse bi-variée. Le test de Chi carré a été utilisé pour mesurer l'association entre deux variables. Le niveau de significativité du test a été de p<0,05. La force des associations a été estimée grâce aux Odds Ratio (OR).

##### Aspects éthiques et déontologiques

L'autorisation de mener l'étude a été obtenue auprès du comité d'éthique locale de la Faculté de Médecine de l'Université de Parakou. Le consentement éclairé de tous les sujets a été obtenu. Une brève explication de l'enquête et de son but a été donnée aux étudiants.

## Résultats

Au total 230 fiches ont été distribuées. Le nombre de fiches récupérées était de 207 soit un taux de réponse de 90%.

### Caractéristiques socio-démographiques de la population d'étude

L'âge moyen des participants était de 21,77 ans ± 2,66 avec des extrêmes de 16 et 32 ans. Le groupe d'âge le plus représenté était celui de 21 à 24 ans soit 48,79% de l'échantillon. Le sexe ratio (H/F) était de 1,76 avec 63,77% des étudiants qui étaient de sexe masculin. La majorité des étudiants était célibataire (85,99%). Les étudiants de nationalité béninoise étaient majoritaires (87,92%). Parmi les 25 étrangers, on distinguait 20 camerounais, deux togolais, deux ivoiriens et un tchadien. La plupart des étudiants vivaient en location (80,68%) dont 4,83% en cité universitaire. Les étudiants étaient répartis respectivement en fonction de leur année d'étude: 41,55% en PCEM2, 29,95% en DCEM2 et 28,50% en DCEM4. Plus de la moitié des étudiants avait redoublé au moins une fois (51,69%). Trente virgule quartre-vingt-deux pourcent des étudiants disposaient d'une bourse d'étude. Les langues maternelles dominantes étaient le Fon et dérivés (43,48%) (Tableau 1).

**Tableau 1:** Caractéristiques socio-démographiques de la population d’étude, Faculté de Médecine de Parakou, 2018

Sexe	Effectif	%
Masculin	132	63,77
Féminin	75	36,23
**Classe d’âge**		
16 – 20	80	38,65
21 - 24	101	48,79
³ 25	26	12,56
Nationalité*		
Béninoise	182	87,92
Autres	25	12,08
**Année d’étude**		
PCEM2	86	41,55
DCEM2	62	29,95
DCEM4	59	28,50
**Statut matrimonial**		
Célibataire	178	85,99
Autres	29	14,01
**Financement de leurs études**		
Boursiers	64	30,92
Autres	143	69,08
**Lieu de résidence**		
Location	157	75,85
Cité universitaire	10	4,83
Autres	40	19,32

### Prévalence des comportements de maltraitance

**Figure 1:**
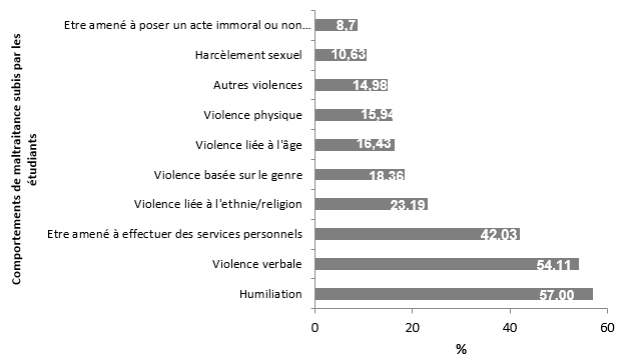
Prévalence des comportements de maltraitance subis par les étudiants de la Faculté de Médecine de Parakou, 2018

La totalité (100%) des enquêtés a subi au moins un comportement de maltraitance depuis son entrée à la FM/UP. Les comportements de maltraitance les plus subis par les étudiants étaient l'humiliation (57%), la violence verbale (54,11%) et le fait d'être amené à effectuer des services personnels (42,03%). Les comportements de maltraitance les moins subis étaient le harcèlement sexuel (10,63%) et le fait d'être amené à poser un acte immoral ou non conforme à l'éthique médicale (08,70%). La Figure 1 montre la prévalence des comportements de maltraitance subis par les étudiants de la FM/UP.

### Fréquence de survenue des comportements de maltraitance

Les étudiants subissaient fréquemment un comportement de maltraitance dans 34,34% des cas, parfois dans 57,83% des cas et rarement dans 7,83% des cas.

### Les auteurs des comportements de maltraitance

Les auteurs les plus fréquents d'humiliation présentés dans le Tableau 2 étaient respectivement les médecins hospitaliers/enseignants (24,76%) et les infirmiers (22,86%). Les auteurs les plus fréquents de violence verbale étaient les médecins hospitaliers/enseignants (24,36%) et les infirmiers (22,49%). Le fait d'être amené à effectuer des services personnels était dû en majorité aux internes/résidents (34,09%). Le harcèlement sexuel était pratiqué de façon équitable par les médecins/enseignants (25%), les infirmiers (25%), les internes (25%) et les camarades étudiants (25%). Les étudiants étaient, eux-mêmes, les principaux auteurs des autres formes de violence subies par leurs camarades à savoir: la violence physique (53,85%), la violence liée au genre (46,88%), la violence liée à l'âge (43,48%), la violence liée à l'ethnie et/ou la religion (46,58%).

**Tableau 2:** Facteurs associés à l’humiliation et aux services personnels, Faculté de Médecine de Parakou, 2018

Humiliation				Services personnels		
Âge	OR	IC	p	OR	IC	p
16-20	1			1		
21-24	3,11	1,69-5,73	0,0003*	2,12	1,15-3,89	0,0157*
25	5,27	1,9-14,57	0,0014*	1,29	0,52-3,25	0,5777
**Année d’étude**						
PCEM2	1			1		
DCEM2	6,28	3,03-13,01	<0,0001*	5,63	2,75-11,51	<0,0001*
DCEM4	7,02	3,31-14,91	<0,0001*	2,44	1,19 - 4,97	0,0141*
**Sexe**						
Masculin	1			1		
Féminin	0,61	0,34-1,08	0,0939	0,80	0,45-1,43	0,4604
**Bourse**						
Oui	1			1		
Non	1,15	0,63-2,09	0,6465	0,76	0,41-1,39	0,3777
**Redoublement**						
Oui	1			1		
Non	0,92	0,53-1,60	0,7799	0,85	0,49-1,48	0,5788

### Rapportage des comportements de maltraitance

Au total 202 étudiants soit 97,58% n'ont pas rapporté les comportements de maltraitance dont ils ont été l'objet. Les principales raisons de non signalement des comportements de maltraitance étaient: le fait que les étudiants n'avaient pas jugé l'incident important pour le dénoncer (38%), et la peur des représailles (24%). Parmi les cinq étudiants qui avaient dénoncé les mauvais comportements subis, deux l'avaient rapporté à un responsable étudiant, deux autres à un enseignant de la faculté, et le dernier à un personnel administratif de la FM/UP.

### Facteurs associés aux comportements de maltraitance

L'humiliation, la violence verbale, le fait d'être amené à effectuer des services personnels, la violence liée à l'ethnie/la religion, et le fait de poser un acte immoral ou contraire à l'éthique médicale étaient significativement associés à l'âge et à l'année d'étude des étudiants (Tableau 2, Tableau 3, Tableau 4). En référence les étudiants dont l'âge était compris entre 16 et 20 ans, les étudiants âgés de 21 à 24 ans avaient trois fois plus de risque de se faire humilier (p=0,0003) et les étudiants âgés de 25 ans et plus avaient cinq fois plus de risque de se faire humilier (p=0,0014). En référence, les étudiants en PCEM2 et les étudiants en DCEM2 avaient six fois plus de risque de se faire humilier (p<0,0001), et les étudiants en DCEM4 avaient sept fois plus de risque de se faire humilier (p<0,0001). Plus les étudiants étaient âgés plus ils subissaient la violence verbale. Il en était de même pour l'année d'étude, plus leur niveau d'étude était élevé plus ils étaient agressés verbalement. Ainsi en référence, les étudiants dont l'âge étaient compris entre 16 et 20 ans, les étudiants âgés de 21 à 24 ans étaient trois fois plus violentés verbalement (p=0,0002) et ceux âgés de 25 et plus subissaient deux fois plus d'agression verbale (p=0,0348). De même, les étudiants en DCEM2 et en DCEM4 subissaient respectivement trois fois plus (p= 0,0009) et cinq fois plus (p<0,0001) de violence verbale que leurs camarades en PCEM2. Les étudiants âgés de 21 à 24 ans étaient plus sollicités pour accomplir des services personnels (deux fois plus) que leurs camarades dont l'âge était compris entre 16 et 20 ans (p=0,0157). Les étudiants inscrits en DCEM2 étaient cinq fois plus sollicités pour effectuer des services personnels que les PCEM2 (p<0,0001), ceux en DECM4 étaient deux fois plus sollicités pour les services personnels que leurs camarades inscrits en PCEM2 (p=0,0141). Les étudiants âgés de 21 à 24 ans avaient deux fois plus de risque de subir une violence liée à l'ethnie ou à la religion que leurs camarades dont l'âge était compris entre 16 et 20 ans (p=0,0360). Ceux âgés de 25 ans et plus avaient quatre fois plus de risque de subir le même type de violence (p=0,0083). Les étudiants en DCEM2 (p=0,0053) et DCEM4 (p=0,0033) subissaient trois fois plus de violence liée à l'ethnie/religion que leurs camarades en PCEM2. Les étudiants âgés de 21 à 24 ans couraient cinq fois plus de risque d'être contraints à poser un acte immoral ou contraire à la déontologie médicale que leurs camarades dont l'âge était compris entre 16 et 20 ans (p=0,0239). Les étudiants en DCEM4 étaient sept fois plus contraints à poser un acte immoral ou contraire à l'éthique médicale que leurs camarades en PCEM2 (p=0,0020). Le harcèlement sexuel était significativement associé au sexe. Les étudiantes avaient trois fois plus de risque de subir un harcèlement sexuel que leurs camarades du sexe opposé (p=0,0069) (Tableau 4). La violence physique, la violence basée sur genre, la violence basée sur l'âge et les autres formes de violence basée sur vos croyances personnelles ou caractéristiques personnelles autre que votre genre, votre race, votre origine ethnique ou votre âge, n'étaient associées à aucune des caractéristiques socio-démographiques des étudiants.

**Tableau 3:** Facteurs associés à la violence verbale et à la violence basée sur l’ethnie, la race et/ou la religion, Faculté de Médecine de Parakou, 2018

Violence verbale				Violence basée sur l’ethnie/la race et /ou la religion		
**Âge**	OR	IC	p	OR	IC	p
16-20	1			1		
21-24	3,14	1,71-5,78	0,0002*	5,76	1,26-26,33	0,0239*
25	2,66	1,07-6,63	0,0348*	5, 08	0,80-32,30	0,0846*
**Année d’étude**						
PCEM2	1			1		
DCEM2	3,16	1,60-6,24	0,0009*	0,93	0,15-5,68	0,9310
DCEM4	5,01	2,42-10,35	<0,0001*	7,82	2,12-28,86	0,0020*
**Sexe**						
Masculin	1			1		
Féminin	0,68	0,38-1,20	0,1847	0,09	0,01-0,70	0,0214*
**Bourse**						
Oui	1			1		
Non	1,24	0,68-2,25	0,4744	0,61	0,19-1,94	0,4081
**Redoublement**						
Oui	1			1		
Non	0,54	0,31-0,93	0,0282*	0,73	0,27-1,92	0,5210

* p significatif

**Tableau 4:** Facteurs associés au harcèlement sexuel et au fait d’être amené à poser un acte immoral ou contraire à l’éthique médicale, Faculté de Médecine de Parakou, 2018

Harcèlement sexuel				Acte immoral		
**Âge**	OR	IC	p	OR	IC	p
16-20	1			1		
21-24	0,68	0,25-1,85	0,4479	5,76	1,26-26,33	0,0239*
25	1,88	0,57-6,21	0,3018	5, 08	0,80-32,30	0,0846*
**Année d’étude**						
PCEM2	1			1		
DCEM2	1,04	0 ,34-3,17	0,9387	5,63	2,75-11,51	<0,0001*
DCEM4	1,53	1,19-4,97	0,4236	2,44	1,19 - 4,97	0,0141*
**Sexe**						
Masculin	1			1		
Féminin	3,56	1,41-8,94	0,0069*	0,80	0,45-1,43	0,4604
**Bourse**						
Oui	1			1		
Non	0,82	0,30-2,21	0,6959	0,76	0,41-1,39	0,3777
**Redoublement**						
Oui	1			1		
Non	1,39	0,57-3,4	0,4641	0,85	0,49-1,48	0,5788

* p significatif

## Discussion

L'objectif principal de cette étude était de déterminer la prévalence des comportements de maltraitance subis par les étudiants et les facteurs associés au sein de la FM/UP en 2018. Au terme de notre étude, il a été constaté que tous les étudiants en médecine ont subi au moins une fois un comportement de maltraitance. Les comportements de maltraitance qui prévalaient le plus sont l'humiliation, la violence verbale et le fait d'être amené à effectuer des services personnels. Cependant, environ 10,63% des étudiants de la FM/UP ont subi un harcèlement sexuel. Quatre-vingt-dix-sept virgule cinquante deux pourcent des étudiants n'avaient pas dénoncé ces comportements. Les auteurs des comportements de maltraitance subis par les étudiants étaient les médecins/enseignants, et les infirmiers en ce qui concerne l'humiliation et la violence verbale puis les internes pour le fait d'être amené à effectuer des services personnels. Les autres formes de violence notamment la violence physique, la violence basée sur le genre, la violence basée sur la religion, l'ethnie et/ou l'âge étaient le fait des étudiants eux-mêmes. Les facteurs associés à ces comportements de maltraitance étaient l'âge et l'année d'étude, pour l'humiliation, la violence verbale et le fait d'être amené à effectuer un service personnel. Les étudiants de sexe féminin étaient trois fois plus harcelés sexuellement que leurs camarades du sexe opposé. La prévalence de survenue d'un comportement de maltraitance est égale à 100% dans notre étude. Ce taux relativement élevé est similaire à celui observé en 2008, par Owoaje et coll au Nigeria (98,5%) [8] et en 2010 par Al Shafaee et coll (96,6%) à Oman [13]. Quoiqu'inférieur à notre taux, la prévalence de la maltraitance des étudiants en médecine est tout aussi élevée sur les autres continents. À Sao Paulo au Brésil en 2013, Peres et coll retrouvait un taux de 92,3% [14], aux Etats Unis en 2011, ce taux était de 64% [5], et de 62,5% au Pakistan en 2007 [14]. Dans notre étude, un comportement de maltraitance survenait fréquemment dans 34,34% des cas. Oku et coll, Peres et coll ont observé pratiquement le même pourcentage respectivement en 2010 (38,5%) au Nigeria [15] et en 2013 (30%) au Brésil [16]. Les comportements de maltraitance les plus subis par les étudiants de la FM/UP étaient l'humiliation, la violence verbale et le fait d'être amené à effectuer un service personnel. L'humiliation et la violence verbale sont retrouvées comme les formes de violence les plus subies par les étudiants dans pratiquement toutes les facultés de médecine où une étude similaire a été conduite [1-2,5-6,8-16].

Par contre le harcèlement sexuel était retrouvé à un taux de 10,63% dans notre étude. Ce taux est relativement inférieur à celui de Rautio et coll en 2005, de Owoaje et coll en 2012 au Nigeria et de Al Shaafee et coll en 2010 qui étaient respectivement de 17% [17], 33,8% [8] et 24,1% [13]. Mais ces deux études n’ont inclus que les étudiants en dernière année des études médicales (c’est-à-dire les internes ou les résidents) et leur définition du harcèlement sexuel qui prenait aussi en compte la violence basée sur le genre. Notre taux est cependant plus élevé que celui de Oku et coll qui ont trouvé une prévalence de 1,8% pour le harcèlement sexuel, cette différence ici pourrait être attribuée au fait que cette étude n'a pas inclus les internes car ces dernières sont plus âgées et susceptibles d'être agressées [15]. Les principaux auteurs de comportements de maltraitance étaient les enseignants, les infirmiers et les internes. L'humiliation et la violence verbale sont le fait des enseignants (maîtres de stage) et des infirmiers dans notre étude. C'est le même constat dans pratiquement toutes les facultés de médecine [5,8,10-17]. Les internes étaient plus impliquées dans le fait de demander à leurs autres camarades des services personnels, car dans notre système hospitalier, les internes jouent un rôle prépondérant dans la prise en charge des patients et détiennent une certaine autorité tacitement reconnue après les médecins responsables de stage. Nous avons observé une association statistiquement significative entre l'âge et l'année d'étude avec les formes de comportements de maltraitance tels que l'humiliation, la violence verbale, la violence basée sur l'ethnie/la race/la religion, le fait d'être amené à effectuer un service personnel et de poser un acte immoral ou contraire à l'éthique médicale. Les étudiants inscrits en deuxième cycle des études médicales et âgés de plus de 21 ans sont plus sujets à la maltraitance. Ceci s'explique par le fait que les stages cliniques débutent généralement au cours de cette période, par conséquent le contact est plus fréquent entre les étudiants en médecine et leurs enseignants d'une part et entre les étudiants en médecine et les infirmiers d'autres part. Ce constat est le même dans la plupart des études [5,8,10-18]. Dans la présente étude, 97,52% des étudiants n'ont pas rapporté les mauvais traitements dont ils ont été l'objet. Ce taux est superposable à celui observé par Ahmadipour (92%) en Iran [19]. En effet la maltraitance est perçue par les étudiants comme faisant partie du processus d’apprentissage, elle est donc perpétuée de façon séculaire. Dans nos facultés, il n'existe pas de structure permettant l'écoute et la dénonciation de façon anonyme cet état de chose d'où la peur des représailles.

### Limites de l'étude

Le questionnaire utilisé est une partie de celui utilisé par l'AAMC pour évaluer la qualité de la formation dans les écoles de médecine américaines. Ce questionnaire a été adapté au cadre de l'étude. Nous avons donc assuré les conditions optimales dans la conception du protocole de cette recherche. Néanmoins, les questions posées étaient sensibles et remontaient à des évènements survenus dans le passé. Les biais d'information ne pouvaient donc pas être entièrement écartés. Notre étude n'a pas pris en compte les conséquences psychosociales de cette maltraitance subie par les étudiants de la FM/UP.

## Conclusion

La maltraitance des étudiants est une réalité à la FM/UP en 2018. Tous les étudiants inclus ont subi au moins un comportement de maltraitance. Les comportements de maltraitance les plus subis sont l'humiliation, la violence verbale et le fait d'être amené à effectuer des services personnels. Les auteurs de ces comportements de maltraitance sont les enseignants, les infirmiers et les étudiants entre eux-mêmes. La majorité des étudiants n'ont pas dénoncé les mauvais comportements dont ils ont été l'objet car cette maltraitance était perçue comme faisant partie intégrante de la formation et aussi à cause de la peur des représailles. Cette étude devrait permettre au conseil pédagogique de la FM/UP de prendre des mesures afin de faciliter la dénonciation des comportements de maltraitance subis par les étudiants.

### Etat des connaissances actuelles sur le sujet

- Maltraitance subie par les étudiants en médecine;
- Sa prévalence élevée;
- Les responsables des mauvais traitements infligés aux étudiants.

### Contribution de notre étude à la connaissance

- Prévalence de la maltraitance des étudiants à la Faculté de Médecine de Parakou (Bénin) en 2018;
- Les facteurs associés à la maltraitance des étudiants à la Faculté de Médecine de Parakou (Bénin) en 2018;
- Mise en œuvre d'action préventive pour réduire le phénomène à la Faculté de Médecine de Parakou.

## Conflits d’intérêts

Les auteurs ne déclarent aucun conflit d'intérêts.
